# An RRM–ZnF RNA recognition module targets RBM10 to exonic sequences to promote exon exclusion

**DOI:** 10.1093/nar/gkx225

**Published:** 2017-04-04

**Authors:** Katherine M. Collins, Yaroslav A. Kainov, Evangelos Christodolou, Debashish Ray, Quaid Morris, Timothy Hughes, Ian A. Taylor, Eugene V. Makeyev, Andres Ramos

**Affiliations:** 1 Institute of Structural and Molecular Biology, University College London, London, WC1E 6BT, UK; 2 Centre for Developmental Neurobiology, King's College London, London SE1 1UL, UK; 3 Structural Biology Science Technology Platform, The Francis Crick Institute, London NW1 1AT, UK; 4 Donnelly Centre, University of Toronto, Toronto M5S 3E1, Canada; 5 Macromolecular Structure Laboratory, The Francis Crick Institute, London NW1 1AT, UK

## Abstract

RBM10 is an RNA-binding protein that plays an essential role in development and is frequently mutated in the context of human disease. RBM10 recognizes a diverse set of RNA motifs in introns and exons and regulates alternative splicing. However, the molecular mechanisms underlying this seemingly relaxed sequence specificity are not understood and functional studies have focused on 3΄ intronic sites only. Here, we dissect the RNA code recognized by RBM10 and relate it to the splicing regulatory function of this protein. We show that a two-domain RRM1–ZnF unit recognizes a GGA-centered motif enriched in RBM10 exonic sites with high affinity and specificity and test that the interaction with these exonic sequences promotes exon skipping. Importantly, a second RRM domain (RRM2) of RBM10 recognizes a C-rich sequence, which explains its known interaction with the intronic 3΄ site of NUMB exon 9 contributing to regulation of the Notch pathway in cancer. Together, these findings explain RBM10's broad RNA specificity and suggest that RBM10 functions as a splicing regulator using two RNA-binding units with different specificities to promote exon skipping.

## INTRODUCTION

RBM10 is an RNA regulator that plays a key role in organismal development and regulation of cell proliferation. Point mutations and deletions in the *RBM10* gene are frequently found in patients with the TARP syndrome (Talipes equinovarus, atrial septal defect, Robin sequence and persistent left superior vena cava), an X-linked inherited pathology associated with malformation of multiple organs and significant early-life mortality (1–3). Additionally, the protein is important for the RNA metabolism of genes associated with palate morphogenesis and with the oral facial digital syndrome (4). Recently, RBM10 was identified as one of the most frequently mutated genes in lung cancer (5,6) and RBM10 mutations have been linked to pancreatic cancer (7) and colorectal carcinoma (8). The high incidence of RBM10 mutations in cancer suggests that they might contribute to pathogenesis of this disease. In line with these reports, RBM10 has been shown to modulate cancer cell proliferation *in vitro* (9,10) and tumour growth in an *in vivo* xenograft model (11).

Recent studies have implicated RBM10 as a splicing regulator for a large set of RNA transcripts (9,10). Knockdown and over-expression experiments followed by transcriptome-wide analyses and combined with the RNA binding landscape of the protein (4,9,10), suggested that a predominant activity of RBM10 in this context is repression of cassette exons containing relatively weak 5΄ and 3΄ splice sites (9,10). Minigene assays have confirmed that RBM10 blocks inclusion of exon 9 of the NUMB gene by binding to an RNA region in the proximity of the branch site of the preceding intron (9) and that recruitment of RBM10 to intronic sites downstream of a cassette exon also promotes its skipping (10). These molecular assays have focused on RBM10's interaction with intronic sites, although exonic sequences account for up to 39% of RBM10 PAR-CLIP clusters (from data in (10)) and presently the functional significance of exonic recruitment of RBM10 remains unknown.

Different models have been put forward to explain how RBM10 can inhibit exon inclusion. In a first model, RBM10 was proposed to interfere with recognition of the splice site by constitutive components of the splicing machinery. For example, skipping of NUMB exon 9 could occur as a result of blocking the binding of the splicing factor U2AF (9). RBM10 was also proposed to interact with intronic sequences to promote skipping of an adjacent cassette exon while simultaneously stimulating the splicing reaction between the upstream and the downstream constitutive exons (10). A more complex type of interaction has been proposed in a recent study where RBM10 has been shown to cross-link not only with mRNA but also with spliceosomal RNAs. This has suggested that RBM10 function may be mediated by its physical interaction with the core splicing machinery (4).

**Figure 1.**
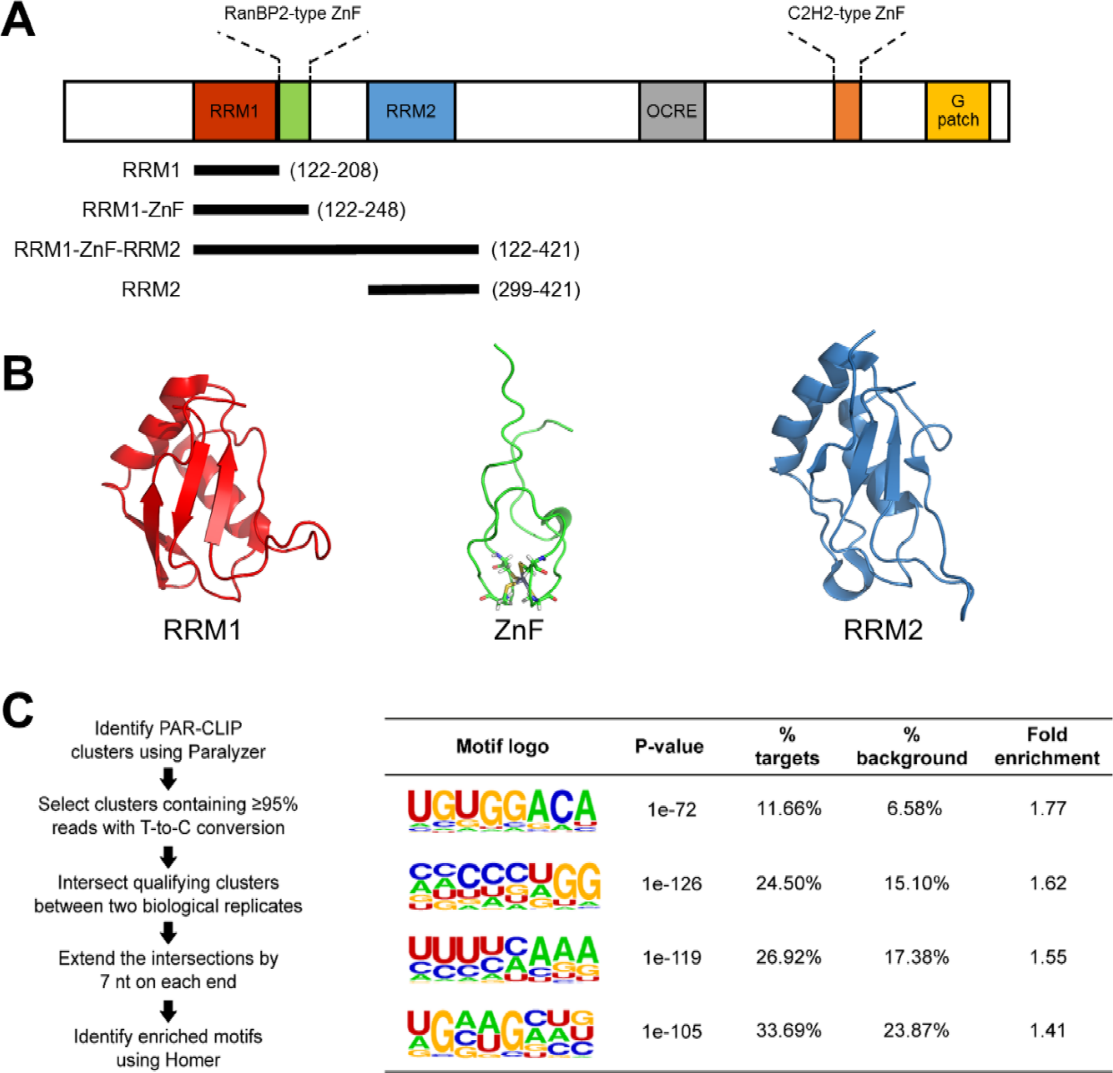
The RNA binding domains of RBM10 and determined binding motifs. (**A**) Domain structure of RBM10 with construct boundaries used in this study displayed below. RNA recognition motif (RRM), RanBP2-type zinc finger (RanBP2 ZnF), C2H2-type zinc finger (C2H2 ZnF, and a G-patch domain (G-patch). (**B**) Ribbon representation of RBM10 RRM1 (PDB: 2LXI) (left), RBM10 RanBP2-type ZnF (PDB: 2MXV) (middle) and RBM10 RRM2 (PDB: 2M2B) (right). (C) The workflow used to identify high-confidence motifs (left) and the top four binding motifs determined for full-length RBM10 (right).

RBM10 contains four classical RNA-binding domains, two RNA recognition motifs (RRMs) and two zinc fingers (ZnFs) (Figure 1A). Three of these domains, RRM1, RanBP2-type ZnF and RRM2, whose individual structures have been recently deposited in the PDB (Figure 1B, accession codes: 2LXI, 2MXV and 2M2B respectively) are sequentially positioned in the N-terminal part of RBM10 creating a long RNA recognition region (Figure 1A) and deletions and mutations of these domains have been linked to cancer (6,9,12). However, the mechanism of RBM10 selection of RNA targets and the positional requirements for the protein to achieve a functional interaction are unclear whilst computational analysis of the RNA-binding landscape of RBM10 has yielded a diverse set of enriched sequence motifs (4,9,10).

It is possible that RBM10 encodes a very low RNA-binding specificity and that this makes the protein a non-discriminant RNA binder. However, RRMs and ZnF are typically sequence-specific domains and we reasoned that the difficulties in identifying RBM10's sequence selectivity might stem from the distinct specificities of the different domains, which could be used to target varied sets of RNAs. We now provide a combination of biophysical, molecular and bioinformatics data to show that RBM10 possesses two distinct RNA recognition units with very different sequence preferences. We propose that this expands the repertoire of RBM10 splicing targets by facilitating its interactions with naturally dissimilar intronic and exonic sequences.

## MATERIALS AND METHODS

### Plasmids for protein production

The gene encoding human RBM10 (Uniprot P98175) was purchased from OriGene (http://www.origene.com). Primers were designed using the Crystallization Construct Designer Software (https://xtal.nki.nl/ccd/) and used to amplify regions of the RBM10 gene while introducing flanking ligation independent cloning sites. The details of constructs used throughout the study are shown in Figure 1. Ligation independent cloning (LIC) as described in (Novagen User Protocol TB453) was used to insert the RBM10 regions into modified pET vectors pET-5247 or pET-52SUMO to produce HRV 3C cleavable amino-terminal His_6_ fusion proteins. DNA sequencing confirmed all plasmid insert sequences prior to recombinant protein expression.

### Protein expression and purification

Proteins were expressed as His_6_ tag or His_6_SUMO3 fusion proteins in *Escherichia coli* BL21(DE3) cells grown to an OD_600_ of 0.6 before the temperature was reduced to 22°C and protein expression was induced with Isopropyl β-D-1-thiogalactopyranoside (IPTG) at a final concentration of 0.5 mM. Cells were lysed and the fusion proteins purified from the soluble fraction of lysate by nickel-affinity chromatography. HRV 3C protease was used to cleave the His_6_ tag or His_6_SUMO3 tag by incubation overnight at 4°C then both the tags and protease were separated from the cleaved protein by nickel-affinity chromatography. The protein was purified further on a HiTrap Heparin HP 5ml column (GE Healthcare) to remove nucleic acid contamination and a final purification step of size exclusion chromatography was performed using an ÄKTA purifier system (GE Healthcare) with a Hiload S75 16/60 Superdex prep grade column (GE Healthcare).

Fractions of pure protein were pooled and concentrated before being dialyzed into a final buffer of 10 mM phosphate pH 6.9, 50 mM NaCl, 1 mM TCEP, 10 μM ZnCl_2_ and concentrated in a Vivaspin MWCO 10 000. Protein concentration was determined from the absorbance at 280 nm.

### NMR spectroscopy

All NMR experiments were carried out using Bruker Avance NMR spectrometers operating at 600, 700, 800 and 950 MHz, ^1^H frequency. Data were processed using the NMRPipe/Draw suite of programs and analyzed using CCPN.

### Backbone assignment

Samples were at ∼400 μM protein concentration in 10 mM sodium phosphate (pH 6.9), 50 mM NaCl, 1 mM TCEP, 10 μM ZnCl_2_. For RBM10 RRM1–ZF protein backbone assignments for ^1^HN, ^15^N, ^13^Cα and ^13^Cβ and ^13^C’ chemical shifts were obtained from HNCACB, CBCA(CO)NH, HNCO and HN(CA)CO experiments. For RBM10 RRM2 protein backbone assignments for ^1^HN, ^15^N, ^13^Cα and ^13^Cβ chemical shifts were obtained from HNCA and CBCA(CO)NH experiments.

### Relaxation

Relaxation data were recorded for RBM10 RRM1–ZF-RRM2, RBM10 RRM1–ZF and RBM10 RRM1–ZF in complex with the RNA oligomer 5΄-CUGUGGA-3΄ at 298 K. Samples consisted of ∼400 μM protein in 10 mM sodium phosphate (pH 6.9), 50 mM NaCl, 1 mM TCEP, 10 μM ZnCl_2_ with 10% D_2_O added for the lock. In order to determine longitudinal and transverse relaxation rates standard experiments were run (13). For RRM1–ZF–RRM2 T1 data were measured with delays of 10, 100, 250, 500, 750 1000, 1500, 2000 ms, and T2 with delays of 8, 16, 32, 48, 64, 88, 112 and 136 ms. For RRM1–ZF both in the free state and bound to RNA delays of 10, 100, 250, 500, 750, 1000, 1500 and 2000 ms were used for T1 measurements and 8, 16, 32, 48, 64, 88, 112, 136 and 160 ms for T2. All data were processed using NMRPipe/Draw and T1 and T2 relaxation rates were determined using CCPN.

### RNA titrations

All RNAs used in NMR titrations were purchased from Dharmacon, GE Healthcare. 40–100 μM ^15^N-labeled samples of protein in 10 mM sodium phosphate (pH 6.9), 50 mM NaCl, 1 mM TCEP, 10 μM ZnCl_2_ were titrated with unlabeled RNA oligomers. ^1^H–^15^N SOFAST-HMQC spectra were recorded at each titration point at 298 or 310 K.

### SIA

Protein samples of 40 μM ^15^N-labeled RBM10 RRM2 in 10 mM sodium phosphate (pH 6.9), 50 mM NaCl, 1 mM TCEP were prepared and individual RNA pools were added to the ratio of 1:2 as required. ^1^H–^15^N SOFAST-HMQC spectra were recorded for the free protein and for each of the protein–RNA samples at 298 K. Spectra were processed using NMRPipe and analyzed in CCPN. A subset of peaks, which shift in the fast exchange regime upon addition of RNA, was chosen. Shift perturbation for each of the peaks upon addition of each RNA pool was measured. For each position in the binding site, the shifts are normalized to the largest of the four then for each pool the shift values are averages over all the chosen peaks to give the final score.

### Isothermal titration calorimetry

All RNAs for ITC experiments were purchased from Dharmacon GE Healthcare. Binding of RBM10 constructs to the RNA oligomers was measured by ITC using an ITC200 instrument (Microcal Inc. Malvern). Samples were dialyzed into in 10 mM sodium phosphate (pH 6.9), 50 mM NaCl, 1 mM TCEP (10 μM ZnCl_2_ was added if protein constructs contained the ZnF domain). Typically, the sample cell contained RNA at 20 μM and the syringe RBM10 RRM1–ZF at 200 μM or RBM10 RRM2 at 190 μM. Titrations were carried out at 25°C using 19 injections of 2 μl with a delay of 180 s between injections. Areas under the peaks were integrated and fitted by least-square procedures assuming a 1:1 binding model using MicroCal Origin 7 software.

### RNAcompete

The RNA pool generation, RNAcompete pulldown assays, and microarray hybridizations were performed as previously described (14,15). Briefly, RNAcompete experiments employed defined RNA pools that are generated from 244 K Agilent custom DNA microarrays. Pool design is based on a de Bruijn sequence of order 11 that was subsequently modified to minimize secondary structure in the designed sequences and minimize intramolecular RNA cross-hybridization. After these modifications, not every 11-mer is represented but each 9-mer is represented at least 16 times. To facilitate internal data comparisons, the pool is split computationally into two sets: Set A and Set B. Each set contains at least 155 copies of all 7-mers except GCTCTTC and CGAGAAG which are removed because they correspond to the SapI/BspQI restriction site used during DNA template pool generation. A φ2.5 bacteriophage T7 promoter initiating with an AGA or AGG sequence is added at the beginning of each probe sequence in the DNA template pool to enable RNA synthesis. The final RNA pool consists of 241 399 individual sequences up to 41 nucleotides in length (15). The microarray design is detailed in (15) and can be ordered from Agilent Technologies using AMADID# 024519. In order to perform the RNAcompete assay, 20 pmol of GST-tagged RBM10 constructs (RRM1–ZnF or RRM1–ZnF–RRM2) and RNA pool (1.5 nmol) were incubated in 1 ml of Binding Buffer (20 mM Hepes pH 7.8, 80 mM KCl, 20 mM NaCl, 10% glycerol, 2 mM DTT, 0.1 μg/μl BSA) containing 20 μl glutathione Sepharose 4B (GE Healthcare) beads (pre-washed three times in binding buffer) for 30 min at 4°C, and subsequently washed four times for 2 min with Binding Buffer at 4°C. One-sided *Z*-scores were calculated for the motifs as described previously (15).

### Bioinformatics

Published RNA-seq and PAR-CLIP data (10) were downloaded from NCBI GEO database (Accession number: GSE44976). Analysis of alternative splicing was done using ExpressionPlot pipeline (16). PAR-CLIP reads were processed using PARpipe pipeline (https://ohlerlab.mdc-berlin.de/software/PARpipe_119/) based on the PARalyzer tool (17). Two replicates were processed separately and only clusters with the T-to-C conversion >0.95 presented in both replicates were used for further analysis. *De novo* search of enriched motifs was done using the HOMER software (18) and clusters extended by 7 nt on each end. Scrambled sequences of the extended clusters were used as a background in this analysis. Motif enrichment in genomic sequence windows was calculated using AMA software as described (19) using region-specific sequence backgrounds and compensating *P*-values for the GC content of each sequence independently.

### Nucleic acids for cell transfection

A minigene containing TNRC6A exon 7 in its natural context – including partial sequences of exons 6 and 8 and the entire sequences of the two intervening introns – was generated by amplifying the corresponding genomic DNA fragment from human genomic DNA (D3035, Sigma-Aldrich) with High-Fidelity DNA polymerase (KK2101, Kapa Biosystems) and the following primers: 5΄-AATCCTCGAGTCGCAAAATGGAGATTGATG and 5΄-TCTTCAATTGTGGTGGCCAATTTAAAGATGA. The PCR product was digested with XhoI and MfeI (New England Biolabs; underlined sites in the primer sequences) and cloned into the pEGFP-N1 plasmid at the XhoI and MfeI sites replacing the *EGFP* gene. Predicted RBM10 binding motif in the exon 7 was mutated by site-directed mutagenesis using Quikchange protocol and the following primers: 5΄-CCTTCTACTCCAGCCACAACAGCAGATAATGGTACTTCAGCATGG-3΄ and 5΄-CCATGCTGAAGTACCATTATCTGCTGTTGTGGCTGGAGTAGAAGG-3΄. pCMV6-Entry plasmid containing human RBM10 coding sequence with a C-terminal Myc-DDK tag was purchased from OriGene (RC200150). Human RBM10 siRNA mixture was from GE Dharmacon (SMARTpool ON-TARGETplus, L-009065-01-0005).

### Cells

HEK-293T cells were propagated in Dulbecco's modified Eagle's medium (DMEM) with GlutaMax (ThermoFisher Scientific, 31966021) supplemented with 10% fetal bovine serum (FBS, Thermo Fisher Scientific, SV30160.03) and 1× penicillin/streptomycin (Thermo Fisher Scientific, 15140122). Cells were co-transfected with plasmids and siRNAs using Lipofectamine 2000 (Thermo Fisher Scientific, 11668019) as recommended. RNA was extracted using Trizol reagent (Thermo Fisher Scientific, 15596018), reverse-transcribed with SuperScript III (Thermo Fisher Scientific, 18080093) and analyzed by semi-quantitative PCR using as described previously (20). Endogenous TNRC6A splicing was assayed using 5΄-CAGACCAGCAAGCACAGGTA-3΄ and 5΄-TGGTGGCCAATTTAAAGATGAGT-3΄ primers. The following primers were used for minigene-derived transcripts: 5΄-GCTACCGGACTCAGATCTCG-3΄ and 5΄-GTAACCATTATAAGCTGCAATAAACAAG-3΄. PCR products were separated using non-denaturing 1.5% agarose gel electrophoresis and the band intensities were quantified using ImageJ.

### Statistics

All statistical analyses were performed in R. Differences in alternative exon inclusion levels were compared using two-tailed Student's *t*-test. Relative changes in exon inclusion were compared using two-tailed Wilcoxon test.

## RESULTS AND DISCUSSION

### RBM10 recognizes RNA motifs comprising the GGA sequence *in vitro* and *in vivo*

Independent analyses of the RBM10 RNA interactome by CLIP-Seq and PAR-CLIP in human cell lines (9,10,21) have yielded a diverse range of enriched motifs potentially recognized by this protein. The top predictions included CUCUGAACUC and CGAUCCCU (9), as well as shorter sequences with divergent nucleotide compositions, GAAGA, UGGA and CUUC (21). Additionally, a recent iCLIP analysis in a mouse cell line identified a CC-centered motif that is enriched in RBM10 cross-linked RNAs (4). Consistent with these results, our bioinformatic analysis designed to identify motifs highly enriched over the background in the existing PAR-CLIP dataset (Figure 1C and Materials and Methods) yielded several distinct sequences. The most enriched motif identified in this manner, by far, was UGUGGACA (Figure 1C). Interestingly, it shared the same sequence core with the CUGUGGAC motif that was previously extracted from the CLIP-seq dataset ((9); albeit ranking fourth in that analysis). Our next highest ranked hit was CCCCCUGG, which could also be related to the C-rich motifs from all three published CLIP analyses (Figure 1C).

**Figure 2.**
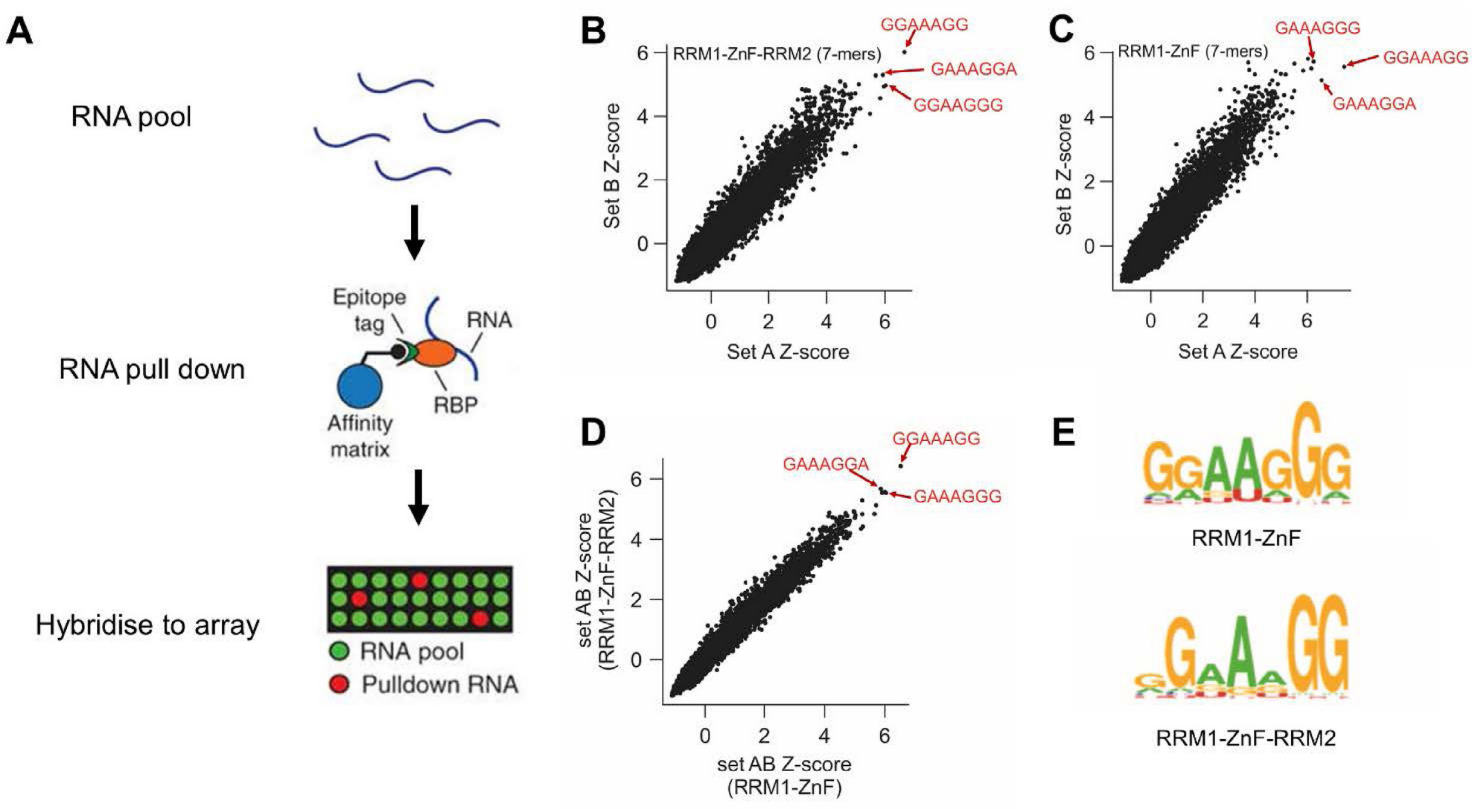
7-mer motifs determined by RNAcompete. (**A**) RNAcompete workflow. (**B** and **C**) Scatterplot of 7-mer *Z*-scores from independent microarray probe sets for RRM1–ZnF–RRM2 (**B**) and RRM1–ZnF (**C**). The sequences of three top-ranking 7-mers are reported in the plots. The GGA sequence is found in two of the three heptamers in each plot and is also found in the top-ranking 7-mers reported in Supplementary Table S1. Here, at least five 7-mers contain the GGA sequence in both assays. (**D**) Scatterplot of 7-mer *Z*-scores from combined A and B probe sets for RRM1–ZnF–RRM2 and RRM1–ZnF. (**E**) Binding motifs determined by RNAcompete for RRM1–ZnF–RRM2 and RRM1–ZnF.

To understand whether the motifs deduced for RBM10 *in vivo* targets related to intrinsic sequence specificity of this protein, we analyzed the binding preference of its main RNA interacting region using RNAcompete (14,15) (Figure 2). Briefly, purified proteins were incubated with a 75-fold excess of a non-randomized RNA pool, RBM10-selected RNAs were identified using microarray analysis and RNA-binding preferences were analyzed computationally (Figure 2A). This uncovered a strong preference of the RRM1–ZnF–RRM2 RNA-binding region for purine-rich heptamers (Figure 2B, D and E), with 5 out of the 10 top-ranking heptamers (Supplementary Table S1) containing the GGA trinucleotide also present in the UGUGGACA and the CUGUGGAC motifs described above. A similar result was obtained when repeating the assay using an RRM1–ZnF-only construct (Figure 2C, E, Supplementary Table S2). Thus, a well-defined GGA sequence is both enriched in the RBM10 RNA interactome and part of RBM10's intrinsic specificity *in vitro*.

### The GGA sequence is recognized by a RanBP2-type ZnF with high specificity

**Figure 3.**
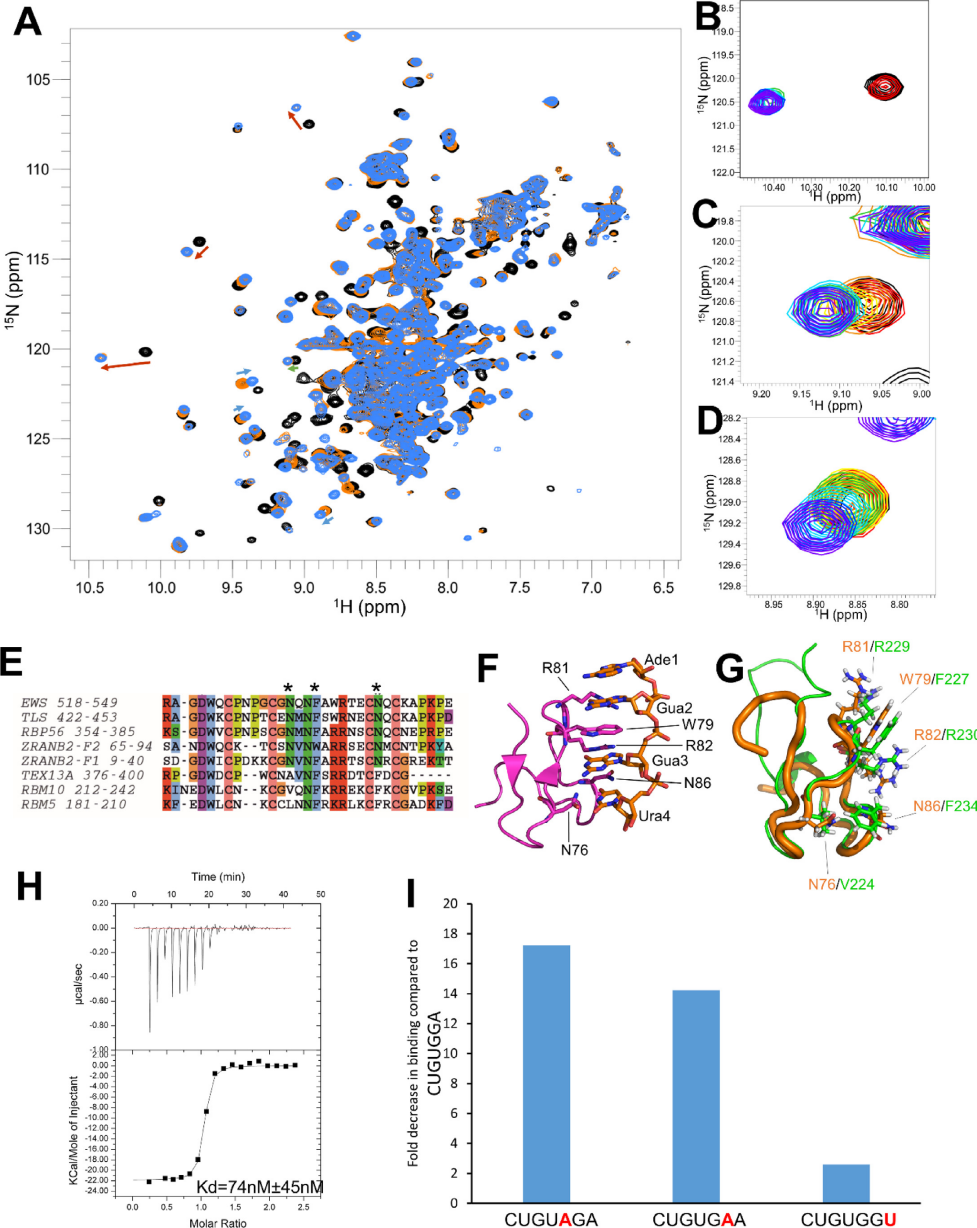
RBM10 binding to the GGA motif.(**A**) Overlay of ^15^N-SOFAST HMQC spectra of RRM1–ZnF–RRM2:CUGUGGA at ratios of 1:0 (black), 1:1 (orange) and 1:4 (blue). Several peaks perturbed upon binding are highlighted with arrows showing the direction of shift: red, RRM1; green, ZnF; blue, RRM2. (**B–D**) Overlay of ^15^N-SOFAST HMQC spectra of RRM1–ZnF–RRM2:CUGUGGA at ratios of 1:0 (black), 1:0.1 (red), 1:0.25 (orange), 1:0.5 (yellow), 1:1 (green), 1:2 (cyan), 1:4 (blue), 1:8 (purple). Magnified views of the chemical shift perturbations of E142 (B), C222 (C), and L350 (D). (**E**) Sequence alignment of the RanBP2-type zinc finger family, coloured using Clustal X color scheme. (**F**) Ribbon representation of ZRAN2-ZF2 (magenta) bound to AGGU (orange) (PDB: 3G9Y). Residues important for the interaction are displayed in stick representation and are highlighted. (**G**) Aligned ribbon representations of free ZRAN2-ZF2 (orange), and RBM10 RanBP2-type ZnF (green). Residues important for RNA interaction are displayed in stick representation and labelled with the residue from ZRAN2–ZF2 in orange and RBM10 RanBP2-type ZnF in green. (**H**) ITC thermogram of interaction between RBM10 RRM1–ZnF and CUGUGGA. Raw data (upper panel), binding isotherm (lower panel). (**I**) Fold decrease in the affinity of binding of the mutated RNAs CUGUAGA, CUGUGAA and CUGUGGU compared to CUGUGGA (data and isotherms in Supplementary Figure S1).

To investigate the molecular basis for GGA specific recognition, we used ^1^H–^15^N correlation NMR spectroscopy to monitor binding of the RRM1–ZnF–RRM2 protein to the CUGUGGA RNA that contains the GGA sequence and is found both in the enriched sequence extracted from the PAR-CLIP data (see above) and the corresponding CLIP-Seq motif (9). Resonances from both the RRM1 and ZnF shift upon RNA binding, which indicates both domains are involved in the interaction with RNA. These resonances are saturated at ∼1 molar equivalent of RNA and many are in a slow exchange regime on a chemical shift timescale, suggesting tight binding with a *K*_d_ in the nM range. In contrast, a second set of peaks are in a fast exchange regime and shift only after the resonances of RRM1 and ZnF are saturated (Figure 3A–D). This shows that a second independent and much weaker binding event, with a *K*_d_ in the μM range, is taking place. The peaks in this set belong to amino acids in the canonical RNA-binding surface of RRM2. This indicates that RRM2 binds RNA using this canonical RNA-binding surface, but its affinity for the CUGUGGA RNA is much lower than the one of the RRM1–ZnF di-domain.

RanBP2-type ZnF domains have been reported to specifically recognize a GGU sequence (Figure 3E–G), and in particular the two G nucleobases present in that sequence (22,23). In these studies, the recognition of GG by ZRANB2-F2 is mediated by a bulky hydrophobic amino acid (W79) and two arginine residues (R81, R82), which are conserved in most RanBP2-type ZnF domain-containing proteins including RBM10 (Figure 3E and G). To test whether RBM10 also recognises a GG dinucleotide within the CUGUGGA RNA, and assess the contribution of the individual nucleobases to motif recognition, we mutated each of the Gs to an A—which is poorly represented in the RNA motif identified in our analysis. We then monitored the affinity changes in the protein–RNA interactions using isothermal titration calorimetry (ITC) (Figure 3H and Supplementary Figure S1). Mutation of either G to A resulted in a 14- to 17-fold reduction in affinity. Further, to test whether RBM10 favors the A of the GGA motif with respect to the canonical U, we measured the affinity changes associated to an A-to-U mutation. The mutation decreased the binding affinity ∼3-fold (Figure 3I). This can be explained by the replacement of two asparagine (N76, N86) residues that make specific contacts with the GGU uracil in the ZRANB2–F2 complex (22) with two large hydrophobic residues, V224 and F234, in RBM10 ZnF (Figure 3G). Our mutational analysis shows that RBM10 ZnF ZRANB2 domain recognizes the GGA sequence with high specificity and that, although the two Gs account for most of the specificity, the final A is also important to maximize binding affinity.

### RRM1–ZnF coupling extends both specificity and affinity of recognition

The RRM1 and ZnF domains are separated by four amino acids and it seems plausible they act together to recognize a UGUGGA or CUGUGGA sequence, with RRM1 interacting with the 5΄ part of this sequence. Residues in the RRM1 and ZnF domains are in the same regime of exchange (Figure 3A–C) and the RNA-mediated chemical shift changes on RRM1 indicate the RNA interacts with the canonical β-sheet surface of the domain (Figure 3A). One question is whether the two domains are physically coupled and act as a rigid two-domain RNA binding unit.

**Figure 4.**
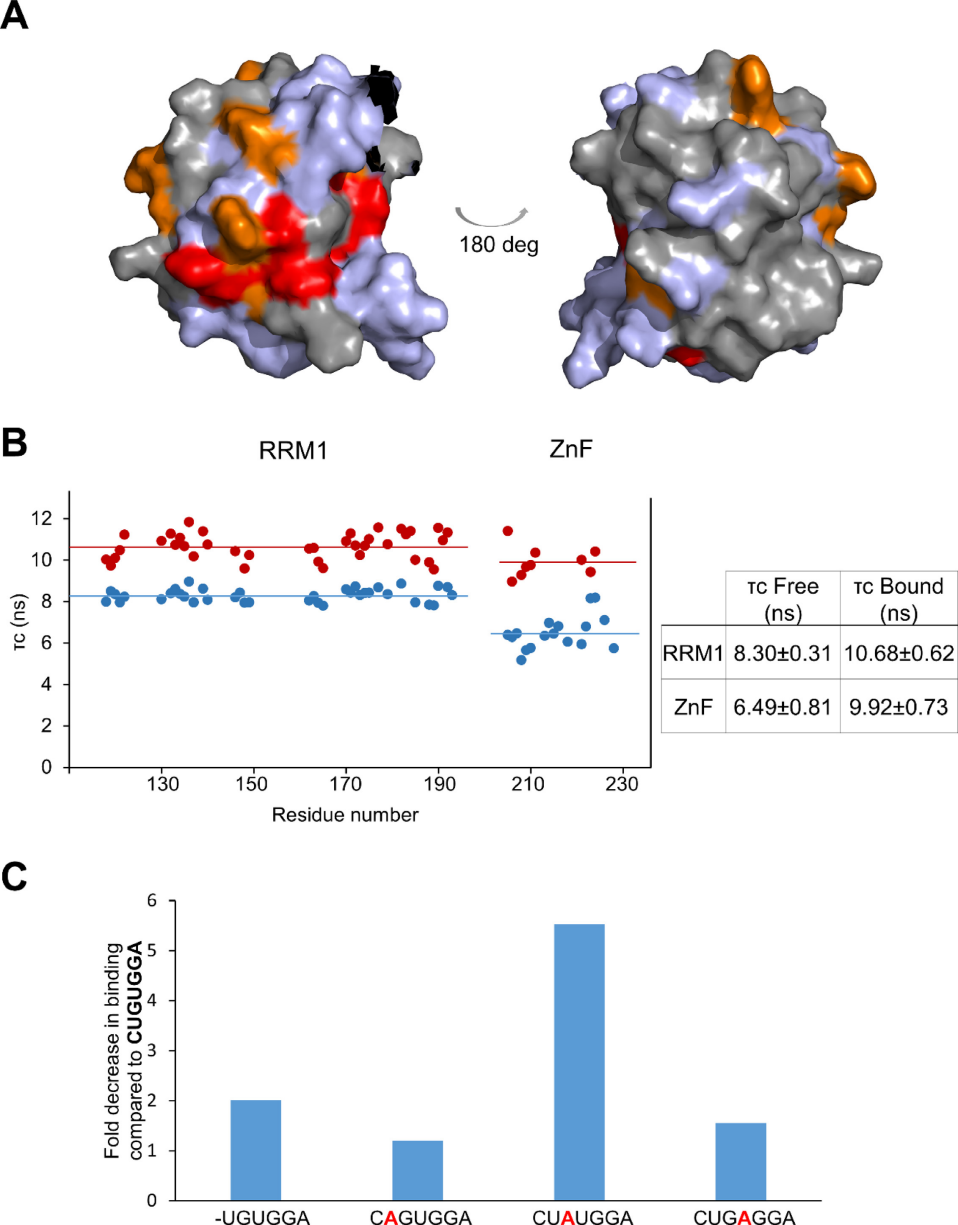
RRM1 and RanBP2-type ZnF interactions and RNA binding. (**A**) Surface representation of RRM1 showing chemical shift perturbations upon unfolding of the ZnF. Greater than average shift perturbation plus one standard deviation (red), greater than average shift perturbation (orange), below average shift perturbation (gray), no data on shift perturbation (blue-grey) (PDB: 2LXI). Left, same orientation as Figure 1B RBM10 RRM1, right 180° rotation. Meaningful changes (red) are limited to a small surface of the domain, consistent with a loose coupling between RRM and ZnF. (**B**) *τ*_c_ of residues in RRM1–ZF in free state (red) and bound to CUGUGGA (blue) and table of *τ*_c_ values. (**C**) Fold decrease in the affinity of binding of the mutated RNAs UGUGGA, CAGUGGA, CUAUGGA and CUGAGGA compared to CUGUGGA (data and isotherms in Supplementary Figure S1).

To address this possibility we made use of two complementary NMR observables, chemical shifts and ^15^N backbone relaxation. These data report respectively on inter-domain contacts and on the hydrodynamic parameters of the two domains. The RRM1 and ZnF domains could be expressed separately and were stable in solution. Only limited changes were visible between the fingerprint ^1^H–^15^N NMR spectra of the RRM1 when in isolation and when expressed together with the ZnF, indicating that the domains do not make extensive interdomain contacts (data not shown). This conclusion is reinforced by the minimal effect on the NMR resonances of RRM1 of unfolding the ZnF, by chelating the Zn ion with EDTA (Figure 4A). Examination of, ^15^N relaxation NMR data confirmed that the two domains re-orient independently in solution. The average rotational correlation time of RRM1 (8.30 ± 0.31 ns) is significantly different from that of the ZnF (6.49 ± 0.81 ns) in the two-domain construct (Figure 4B). However, when bound to CUGUGGA RNA the rotational correlation times of RRM1 (10.68 ± 0.61 ns) and ZnF (9.92 ± 0.73 ns) converge to within the experimental error of each value, indicating the two domains come together to bind one RNA molecule to mediate high-affinity recognition of the longer RNA target sequence (Figure 4B).

Finally, to evaluate to what extent RRM1 contributes to the specificity of recognition we compared the binding of the CUGUGGA and UGUGGA oligonucleotides using NMR and ITC. Removal of the first nucleotide resulted in a very small (2-fold) affinity loss in ITC experiments (Figure 4C) and did not reduce the RNA-binding surface on the protein as assessed by NMR. We then individually mutated each of the UGU nucleotides (positions 2, 3 and 4) to A and measured the changes in affinity using ITC. Mutation of either U (position 2 or position 4) present in the consensus sequence resulted in less than a 2-fold reduction in affinity, while mutation of the G (position 3) resulted in a more significant 5-fold reduction in affinity (Figure 4C). We concluded that the RRM1 and ZnF domains are loosely linked and come together to recognise a 5΄-extended GGA motif, with the GGA element playing a key role in recognition and an additional non adjacent 5΄ G adding to the specificity of the interaction.

### RRM2 recognises a C-rich RNA sequence, explaining RNA interactome motifs and NUMB intronic recognition

Our data has shown that, RBM10 RRM1–ZnF recognition of the (C)UGUGGA sequence is a key element defining the RBM10-RNA interactome. However, RBM10–RNA interactome data indicate that additional RNA sequences are likely recognized by RBM10. Our RNAcompete assays show that the GGA signal is diluted when RRM2 is present in the construct and we reasoned that this domain might facilitate RBM10 interaction with sequences different from those recognized by the RRM1–ZnF module. Although the RRM2 domain of the RBM10 paralogue RBM5 has been shown to bind a diverse range of RNA sequences with similar affinity (24) the sequence specificity of RBM10 RRM2 is still unknown.

**Figure 5.**
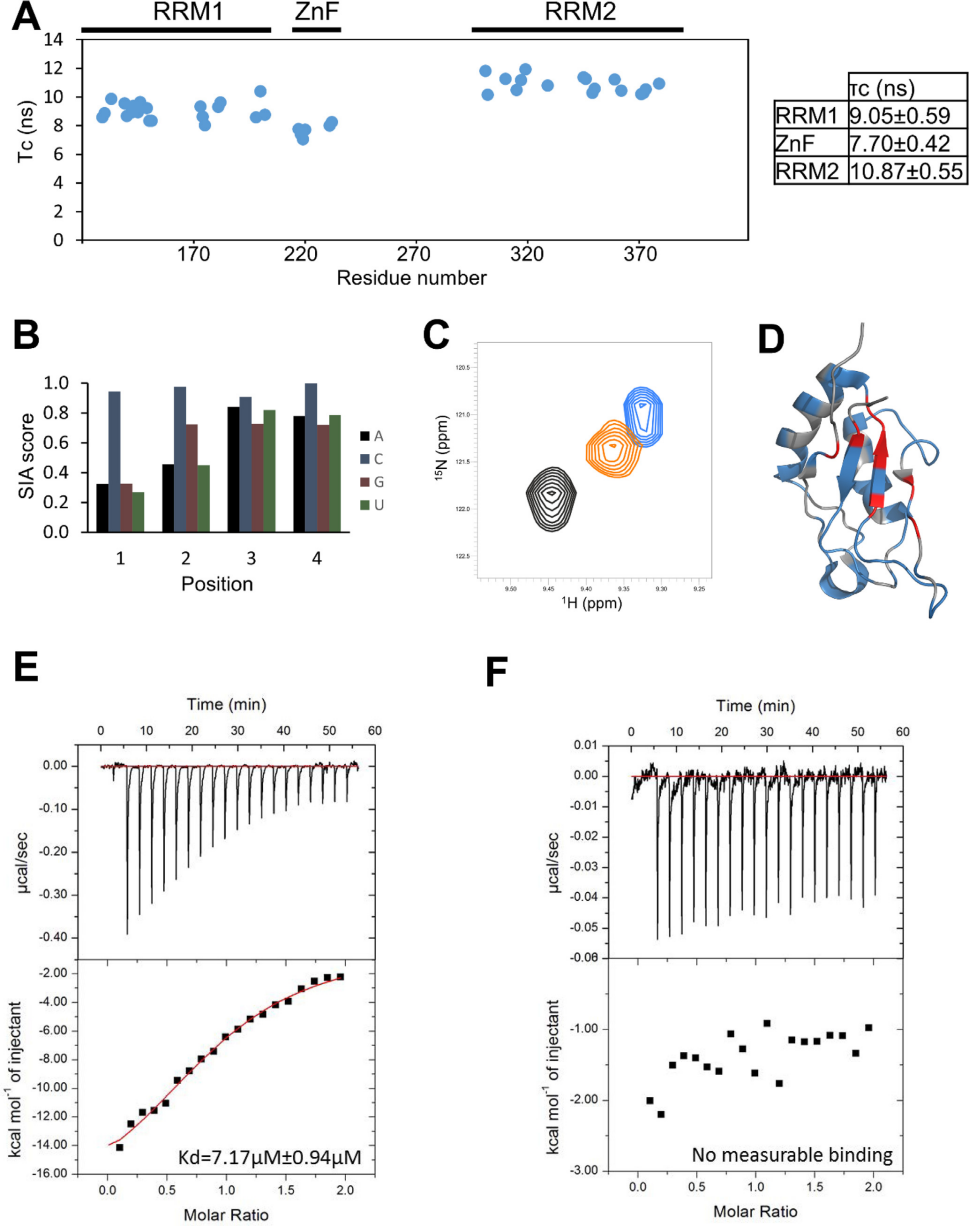
RBM10 RRM2 RNA binding. (A) τ_c_ of residues in RRM1–ZnF–RRM2, with a table of the average τ_c_ values in regions of secondary structure. (B) SIA scores for RRM2. These are comparative scores that reflect the preference of the domain for one nucleobase versus the other. Notice the significant preference of RRM2 to cytosine in positions 1 and 2. (C) Overlay of ^15^N-SOFAST HMQC spectra of RRM2 in the free state (black) and in the presence of NNNN (orange) and CCNC (blue) at ratios of 1:4. (D) Ribbon representation of RRM2 (PDB: 2M2B). Greater than average shift perturbation plus one standard deviation (red), less than average shift perturbation plus one standard deviation (blue), no data on shift perturbation (grey). The RNA binds on the canonical β-sheet surface of the domains. (E-F) ITC thermogram of interaction between RBM10 RRM2 and (E) CCCAC, (F) UAAUA. Raw data (upper panel), binding isotherm (lower panel).

RRM2 is separated from the ZnF domain by a long linker, and the two domains are likely to be structurally decoupled. ^15^N T1 and T2 NMR relaxation data recorded on the RRM1–ZnF–RRM2 construct were used to calculate the rotational correlation times (τ_c_) of the different domains. The τ_c_ of RRM2 is different from that of RRM1, and of the ZnF, and this indicates that the RRM2 re-orients quasi-independently in solution (Figure 5A). Therefore, RRM2 can be considered a structurally independent unit. As no prior information is available on RBM10 RRM2 specificity we used Scaffold Independent Analysis (SIA) (25) to assess the RRM2 nucleobase preference. This assay evaluates the nucleobase preference of a domain in each of the positions of the bound RNA sequence. For each position, the assay compares the molar fraction of bound protein in four different complexes where the same domain is bound to four different RNAs, using NMR. The four RNAs have a fully randomized sequence except for the position to be evaluated. Here, each of the four RNAs has a different fixed nucleobase. The comparison between the molar fractions of bound protein reports on the preference towards each nucleobase. The result of the SIA assay defined a CCNC sequence as the preferred RBM10 RRM2-binding sequence, with most specificity provided by the first two nucleobases (Figure 5B). This result was validated by the larger chemical shift changes we observed in fingerprint NMR spectra when titrating the CCNC RNA into a sample of RBM10 RRM2 with respect to a randomized (NNNN) oligo (Figure 5C). In these assays, the RNA binds on the protein canonical β-sheet surface (Figure 5D). To quantify the binding affinity of the RRM2 domain for a target sequence, we used ITC and a short RNA oligo that contains the SIA motif, CCCAC (Figure 5E). The equilibrium dissociation constant is ∼ 7 μM, indicating a moderate binding affinity. However, ITC also showed that the domains select strongly against other single stranded RNA sequences. Titrating a UAAUA oligo into RRM2 resulted in no appreciable binding (Figure 5F). Further, it is worth considering that, given the repetitive nature of the RRM2 binding sequence the domain is likely to bind with significantly higher apparent affinity to repetitive polyC tracts, because of the high local density of binding sites.

Importantly, the RRM2 specificity preference for C-rich sequences is consistent with the CCCCCUGG motif identified in our bioinformatics analyses of the PAR-CLIP data, the CGAUCCCU motif by Bechara *et al*. and the recently reported CC motif enriched in RBM10 cross-linked RNAs in a mouse cell line (4). Moreover, a pyrimidine-rich intronic 3΄ segment containing runs of Cs but no GGAs has been directly shown to recruit RBM10 and mediate repression of the alternative exon 9 in NUMB pre-mRNA. It is therefore likely that RRM2 RNA specificity accounts for interaction of RBM10 with C-rich elements - such as those present in exon-adjacent intronic sequences proposed to recruit the protein. More generally, our data indicate that the complex RBM10–RNA recognition code is specified by distinct sequence specificities of its constituent RNA-binding domains rather than these domains interacting with largely divergent motifs with low specificity.

### RBM10 sites containing the GGA core are enriched in exons

**Figure 6.**
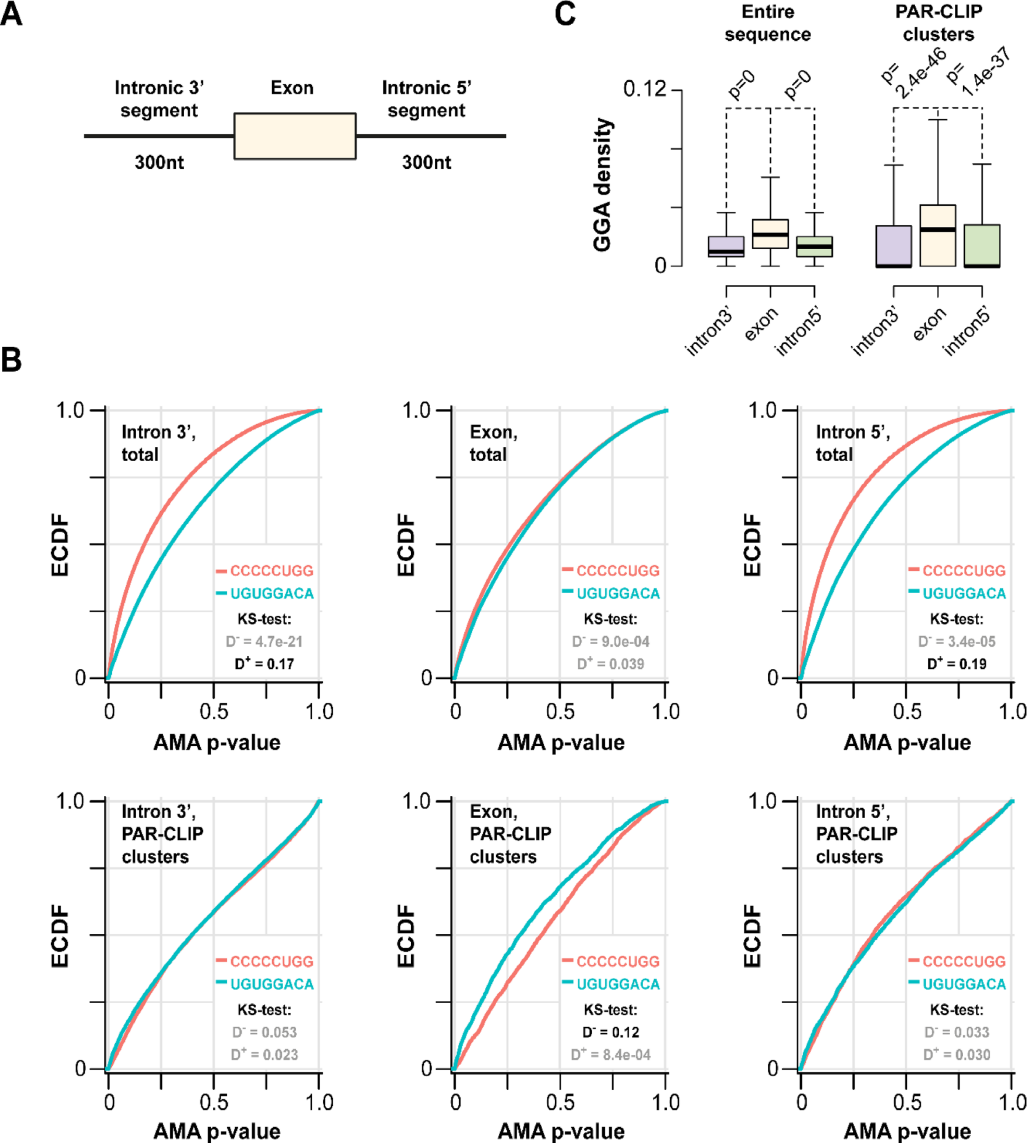
Unequal partitioning of distinct RBM10 motifs between exons and introns. (**A**) Exonic and the adjacent intronic regions used to analyze genome-wide motif occurrence. (**B**) Background-corrected incidence of the UGUGGACA and CCCCCUGG motifs defined as PWMs was compared for the three regions shown in (A) using AMA (19); also see Experimental procedures). Analyses of overall motif incidence in these regions (the top three graphs) showed that CCCCCUGG-like sequences occur more frequently than the UGUGGACA-like ones in intronic 3΄ and 5΄ segments but not in exonic sequences. This was evident from the significant left shift in AMA (average motif affinity) *P*-values for CCCCUGG as compared to UGUGGACA (corresponding single-sided KS-test *D*^+^ values ≥0.17 with *P* < 2.2e–16). By comparison, similar analyses carried out for RBM10-interacting sequences (the bottom three graphs) showed comparable occurrence of the two motifs in the two intronic regions and noticeably higher incidence of the UGUGGACA motif in exons as compared to its CCCCCUGG counterpart (corresponding single-sided KS-test *D*^−^ value 0.12 with *P* = 4.8e–15). (**C**) GGA densities for the three regions defined in (A). This trinucleotide is generally enriched in exons compared to the adjacent intronic sequences (the three box plots on the left). This enrichment is even more apparent for RBM10 PAR-CLIP clusters as evident from the stronger separation of the medians (the three box plots on the right). Data were compared using two-sided Wilcoxon rank sum test.

Given that RRM1–ZnF and RRM2 are two RNA-binding units that recognise two very different sequences, *in vitro* and in the cell, we wondered whether these recognition events could mediate selection of different sets of targets. RBM10 is known to interact with both exons and relatively short (<300 nt) intronic sequences immediately preceding (3΄ segments) and following (5΄ segments) exons (4,9,10) and has been reported to recognize selectively a diverse range of purine-rich and pyrimidine-rich sequences in both exons and introns (21). We therefore compared the relative enrichment of the UGUGGACA and CCCCCUGG motifs defined as corresponding position weight matrices (PWMs) within these genomic regions (Figure 6A and see Materials and Methods). Notably, the overall background-corrected incidence of the CCCCCUGG motif in intronic 3΄ and 5΄ segments was markedly higher than that of UGUGGACA (Figure 6B) even though the two motifs occurred with comparable frequencies in exons (Figure 6B).

When we repeated this analysis for RBM10-interacting sequences, we noticed a general increase in the relative frequency of UGUGGACA-like sequences compared to CCCCCUGG-like sequences for all three regions (Figure 6B). This was possibly a result of the stronger enrichment of the UGUGGACA motifs in the RBM10 interactome (1.77-fold) versus CCCCCUGG (1.62-fold) (Figure 1C). Importantly, this trend resulted in comparable relative frequencies of the two motifs in the intronic regions and a clearly higher relative frequency of UGUGGACA-like sequences in exons (Figure 6B). Consistent with the relative enrichment of the UGUGGACA motif in exonic sequences in general and exonic sequences interacting with RBM10 in particular, the overall density of the GGA trinucleotide, which represents the specific core of this sequence, was significantly higher in exons than in the two exon-adjacent intronic regions (Figure 6C). Interestingly, this difference became even more apparent when we repeated the comparison for exonic and intronic RBM10-interacting clusters (Figure 6C). The distinct intronic/exonic distribution of the two motifs within the RBM10 indicates that the different domains may play different roles in recognition depending on the specific target. It also suggests that a possible role for the GGA specificity of the ZnF is to guarantee access of RBM10 to exonic GGA regions in the proximity of the splice site.

### Recognition of an exonic CUGUGGA sequence mediates splicing regulation of TNRC6A

RBM10 has been reported to control splicing of a large number of alternative exons. However, the only natural splicing target of this protein directly tested so far has been the C-rich intronic sequence preceding RBM10-repressed NUMB exon 9 (9). Similarly, artificial tethering of RBM10 protein to an intronic position downstream of a cassette exon promoted skipping of this exon (10). Given the efficient interaction of RBM10 with exons in general (9,10) and exon-enriched (C)UGUGGA-like sequences in particular (Figure 6), we analyzed whether recruitment of RBM10 to corresponding exonic locations was functionally important.

**Figure 7.**
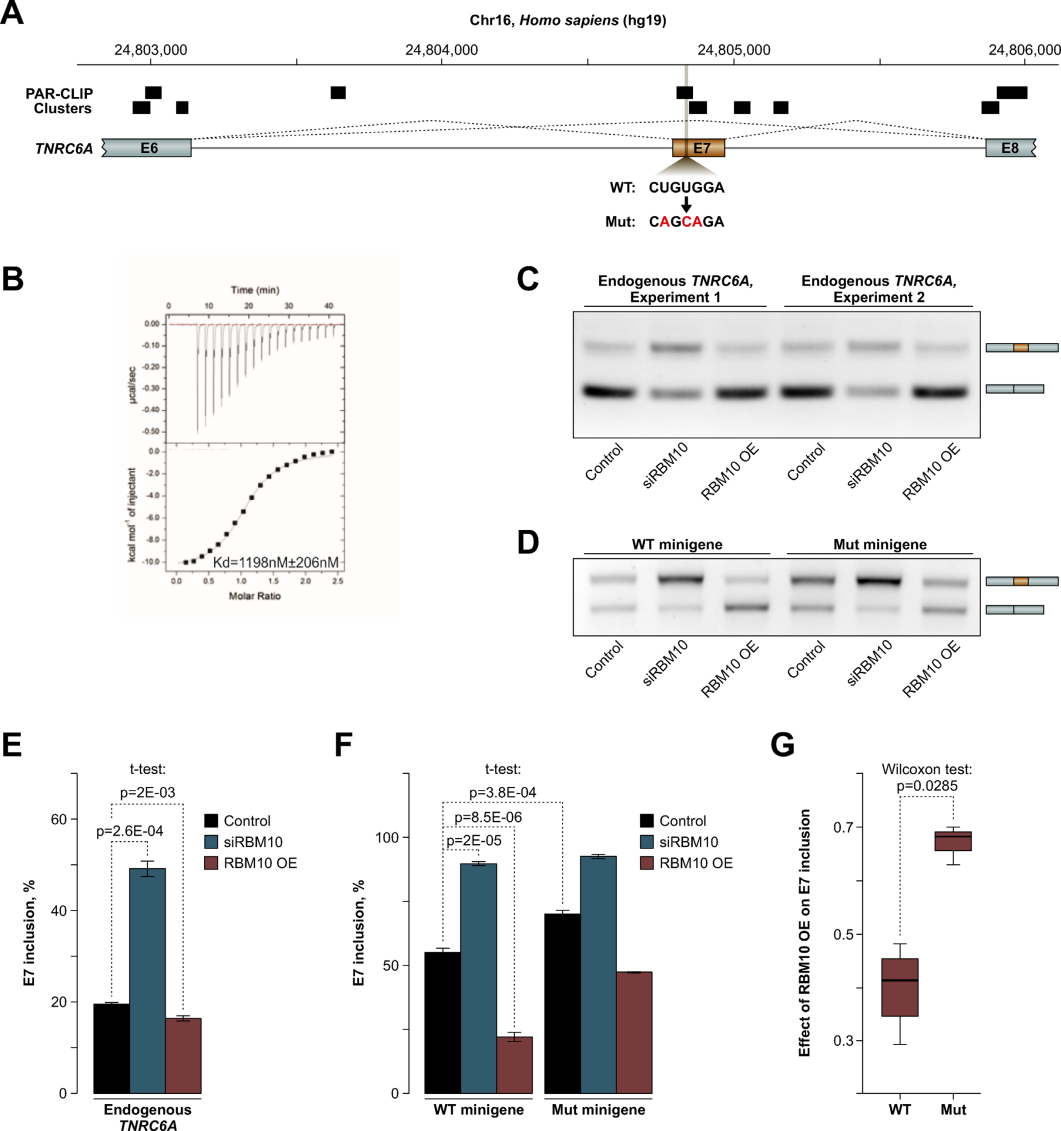
RBM10 binding to an exonic CUGUGGA sequence promotes exon skipping (**A**) Schematic of the regulated part of the TNRC6A pre-mRNA. Positions of RBM10 binding sites identified by PAR-CLIP are shown on the top and the mutated RBM10-specific sequence is indicated at the bottom. (**B**) ITC data recorded during a titration of RBM10–ZnF with CAGCAGA (upper panel) and binding isotherm for the same titration (lower panel). (**C**) HEK293T cells were transfected with RBM10-specific siRNAs (siRBM10) or an RBM10 expression construct (RBM10 OE) and the effects of these treatments on the endogenous TNRC6A exon 7 (E7) were analyzed by RT-PCR. RBM10 knockdown increases E7 inclusion RBM10 over-expression leads to a modest but significant (see Figure 7E) skipping effect. The panel shows the data for two independent transfection experiments. (**D**) HEK293T cells treated as above were additionally transfected with a wild-type or a mutant TNRC6A minigene and the effect of altered RBM10 expression on splicing of minigene-derived transcripts was analyzed by RT-PCR. Note that RBM10 knockdown leads to increased inclusion of TNRC6A E7 whereas RBM10 over-expression promotes skipping. Mutation of the RBM10 recognition motif shifts the splicing pattern toward E7 inclusion. (**E**) Quantitation of the results in (C) presented as PSI values. Data are averaged from four independent experiments with the error bars showing SD. (**F**) Quantitation of the results in (D) as PSI values. Data are averaged from four independent experiments. Error bars show SD. (**G**) Quantitation of the effect of RBM10 overexpression on splicing of the wild-type and the mutant TNRC6A transcripts. Shown are box plots of ratios between E7 PSI values in RBM10 OE and Control samples. Note that compared to their wild-type counterparts transcripts derived form the mutant minigene are significantly less sensitive to changes in the RBM10 levels. Data are averaged from four independent experiments and compared by two-tailed Wilcoxon rank sum test.

Inspection of the list containing high-confidence RBM10 splicing targets (Supplementary Table S3) identified previously unknown RBM10-repressed exon 7 in TNRC6A transcripts encoding a critical component of the microRNA pathway (Figure 7A). This exon contains an exact copy of the CUGUGGA motif overlapping RBM10 PAR-CLIP clusters (Figure 7A). To address functional significance of this element, we assayed the effects of RBM10 knockdown and over-expression in human HEK293T cells on splicing of the endogenous TNRC6A pre-mRNAs as well as minigene-derived transcripts containing TNRC6A exon 7 in its natural context (Figure 7A). In both cases, RNAi-mediated knockdown of RBM10 resulted in increased inclusion of exon 7, whereas over-expression of this protein promoted skipping (Figure 7C–F). Consistent with the repressive activity of RBM10 in this system, mutation of the CUGUGGA sequence to CAGCAGA (interacting with RBM10 RRM1–ZnF 16-fold weaker than CUGUGGA; Figure 7B) increased the basal inclusion level of exon 7 (Figure 7D and F). Importantly, when we compared the ratios between exon 7 ‘percent spliced in’ (PSI) values in the control and the overexpression samples, the mutant exon was noticeably less responsive to RBM10 than its wild-type counterpart (Figure 7G). The remaining regulation of the mutant exon was likely a result of RBM10 interaction with its other sites present in the minigene sequence (see PAR-CLIP track in Figure 7A). Overall these data suggest that RBM10 recruitment to exonic CUGUGGA elements promotes exon skipping.

## CONCLUSIONS

RBM10 is a multi-domain RNA-binding protein with important functions in development and in cancer. RBM10 regulates alternative splicing in a large set of genes but how the protein selects its targets is unclear, and the requirements for its positioning in the proximity of the intron/exon junction are not understood. Here, we show that the RRM1 and ZnF domain of the protein, although not locked in a preformed structural interaction, come together to recognise CUGUGGA-like sequences, with the interaction between the ZnF binding and the GGA unit providing most of the recognition specificity. The specificity towards GGA dominates both the results of our analysis of RBM10–RNA interactome data and our *in vitro* unbiased analysis of the sequence specificity.

In addition, we identify a second C-rich target sequence whose recognition is mediated by the RRM2 domain. This second signal, also found in the RBM10–RNA interactome and shown to be important for regulation, is masked by the RRM1–ZnF signal when evaluating the specificity of the three-domain protein in RNAcompete assays. The interference between RNA modules with different specificities explains, at least in part, the difficulties encountered in defining RBM10's principles of target selection. This study highlights what may be a common a problem in determining the rules of target selection in multi-domain splicing regulators.

While we show that RBM10's multiple RNA-binding domains recognize different RNA sequences, we also question whether the two RNA recognition units described above play a different role in positioning the protein on the RNA. We analyzed the intron/exon partition of RBM10's binding sites and show that the GGA-based motif, but not the C-rich one, is enriched in exonic sites. This suggests the protein uses the high specificity of the ZnF domain to access GGA-rich exonic sequences and overall that the differential action of two RNA recognition units may facilitate efficient targeting of exonic versus exon-adjacent intronic regions. Of note, purine-rich sequences have been previously shown to function as exonic splicing enhancers interacting with cognate SR proteins ((26) and references therein). Future studies should explore the possibility that RBM10 physically or functionally antagonizes splicing activators interacting with overlapping exonic sequences.

Regardless, testing the effect of the RRM1–ZnF interaction on the regulation of TNRC6A splicing using minigene assays, we showed that it results in exon exclusion, similar to the previously reported RBM10 intronic sites. These functional data support the idea of a common action for different RBM10 targets and suggest that the recognition of two diverse sequence motifs may be linked to the ability of this splicing regulator to access RNA regions on either side of the splice site.

## ACCESSION NUMBER

Microarray data were submitted to the GEO database under the accession number GSE96990.

## Supplementary Material

Supplementary Data
