# Supplementary material for: An RRM–ZnF RNA recognition module targets RBM10 to exonic sequences to promote exon exclusion
Source: Nucleic Acids Res. 2017 Apr 4;45(11):6761–74. doi: 10.1093/nar/gkx225 (PMC5499739; doi:10.1093/nar/gkx225)
Supplement: Supplementary Data [file gkx225_Supp.zip › nar-03559-r-2016-File003.pdf]

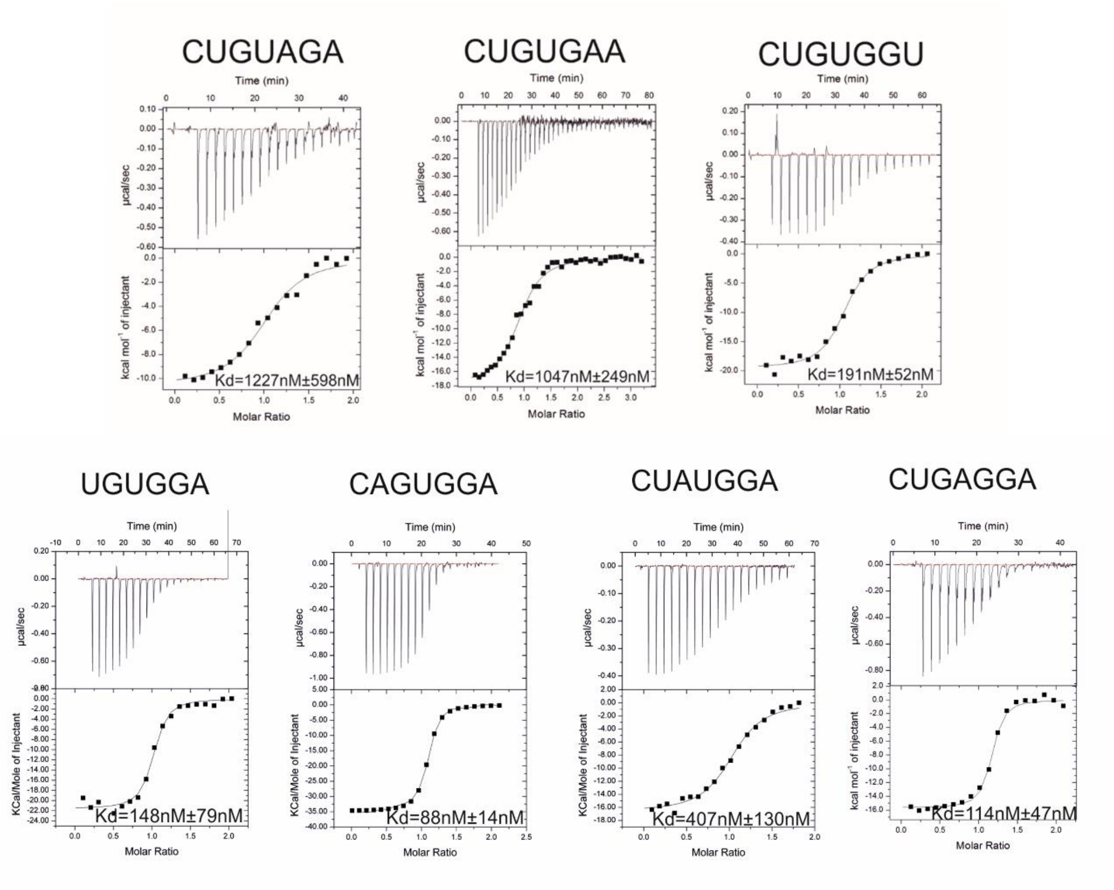

Figure S1

ITC thermogram of interactions between RBM10 RRM1-ZnF and CUGUGGA mutants. Raw data (upper panel), binding isotherm (lower panel). K_{ds} are reported.

Supplementary Tables (provided as separate documents)

Table S1 – Heptamers (7-mers) and enrichment scores (Z-score) in RNAcompete probe sets for RBM10 RRM1-ZnF-RRM2.

Table S2 – Heptamers (7-mers) and enrichment scores (Z-score) in RNAcompete probe sets for RBM10 RRM1-ZnF.

Table S3 – RBM10-regulated cassette exons identified by ExpressionPlot.

Table S4 – T1 and T2 values for RBM10 constructs.
